# Chemotherapy-Induced Peripheral Neuropathy and Changes in Cytoskeleton

**DOI:** 10.3390/ijms20092287

**Published:** 2019-05-09

**Authors:** Alessio Malacrida, Cristina Meregalli, Virginia Rodriguez-Menendez, Gabriella Nicolini

**Affiliations:** School of Medicine and Surgery, Experimental Neurology Unit and Milan Center for Neuroscience, University of Milano-Bicocca, via Cadore 48, 20900 Monza, MB, Italy; alessio.malacrida@unimib.it (A.M.); cristina.meregalli@unimib.it (C.M.); gabriella.nicolini@unimib.it (G.N.)

**Keywords:** chemotherapy-induced-peripheral-neuropathy, cytoskeleton, microfilaments, microtubules, neurofilaments, dorsal root ganglia, neurotoxicity, cytoskeleton-related proteins, crosstalk

## Abstract

Despite the different antineoplastic mechanisms of action, peripheral neurotoxicity induced by all chemotherapy drugs (anti-tubulin agents, platinum compounds, proteasome inhibitors, thalidomide) is associated with neuron morphological changes ascribable to cytoskeleton modifications. The “dying back” degeneration of distal terminals (sensory nerves) of dorsal root ganglia sensory neurons, observed in animal models, in in vitro cultures and biopsies of patients is the most evident hallmark of the perturbation of the cytoskeleton. On the other hand, in highly polarized cells like neurons, the cytoskeleton carries out its role not only in axons but also has a fundamental role in dendrite plasticity and in the organization of soma. In the literature, there are many studies focused on the antineoplastic-induced alteration of microtubule organization (and consequently, fast axonal transport defects) while very few studies have investigated the effect of the different classes of drugs on microfilaments, intermediate filaments and associated proteins. Therefore, in this review, we will focus on: (1) Highlighting the fundamental role of the crosstalk among the three filamentous subsystems and (2) investigating pivotal cytoskeleton-associated proteins.

## 1. Chemotherapy-Induced Peripheral Neuropathy (CIPN)

Chemotherapy-induced peripheral neuropathy (CIPN) is a debilitating and dose-limiting side effect manifested mainly by a sensory, length-dependent process, that results from a drug cumulative dose. CIPN symptoms can be sufficiently severe to require a reduction in drug dosage or discontinuation of treatment [1], causing a significant hindrance to favorable outcomes and long-term patient quality of life. The development of more efficient antitumor therapy has led to a steady increase in the survival rates of patients, with a subset of them who experience chronic CIPN symptoms that continue even after the end of the therapy. As a result, a focus on late toxicity of commonly used antineoplastic drugs has emerged. 

CIPN is associated with many drugs that differ both in their antineoplastic action and in the proposed neurotoxic mechanism. 

CIPN has been reported in patients treated with platinum-based drugs, including cisplatin and oxaliplatin, microtubule-targeting agents (MTA) [2,3] such as taxanes (paclitaxel and docetaxel), vinca alkaloids (particularly vincristine and vinblastine), epothilones and eribulin, and also proteasome inhibitors (bortezomib) and immunomodulatory drugs (thalidomide). 

Although the specific anticancer effect of all classes of drugs is well known, their molecular and cellular impact on the peripheral nervous system is not completely clear. While some classes of antineoplastic drugs have potentially the antiproliferative mechanism strictly linked to their neurotoxicity action (i.e., anti-tubulin compounds), others, for example, the platinum-based drugs, show different neurotoxic effects unrelated to their antineoplastic activity (Table 1).

Below are the classical antitumor mechanisms of action of the different classes of antineoplastic drugs.

- Taxanes interfere with mitotic spindle formation in cancer cells [4,5,6].
- Epothilones exert their effect by stabilizing microtubules which leads to apoptosis in cancer cells [18].
- Eribulin is known to bind at the plus (+) ends of the microtubule, inducing an increase of microtubule depolymerization [16].
- Vinca alkaloid compounds belong to microtubule assembly inhibitor drugs by binding to free tubulin dimers, close to the GTP-binding sites, which induces cell death [13,14].
- Platinum compounds induce adducts of DNA strands and/or proteins, which interfere with cell viability and division in replicating cells [19,20].
- Proteasome inhibitors exert their antiproliferative action by inhibiting the proteasome—the primary intracellular protein degradation machinery—which prevents proteolytic cleavage of intracellular proteins and results in protein aggregate accumulation in tumor cells, leading to cell cycle arrest and apoptosis [22,23].
- Thalidomide mechanism of action is poorly understood but immunomodulation and antiangiogenic effects appear to be involved in its antiproliferative activity, as well as down-regulation of tumor necrosis factor alpha (TNFα) [24].

CIPN in cancer patients generally emerges with various sensory symptoms including paresthesia and dysesthesia, heat/cold hyperalgesia and tingling, which can degenerate into severe sensory and motor damages. Moreover, painful symptoms, such as burning sensation and/or stabbing pain, can occur [25]. Clinically, these manifestations develop in a glove and stocking distribution due to preferential neurotoxic effect on longer axons. Depending on the anti-tumor treatment, the neuropathic symptoms will differ in degree of severity, clinical features and recovery [26]. CIPN current clinical strategies primarily aim to relieve symptoms, focusing on pain.

Due to the lack of knowledge of the pathogenic mechanisms of CIPN, preventive or symptomatic treatments are usually ineffective and neuroprotective agents to prevent or manage CIPN are inadequate. Many efforts have been made to develop strategies of prevention and treatment of CIPN for the recovery of the patient’s condition and quality of life, but at present, there are no established therapeutic strategies to prevent these adverse events [1,27]. Many groups are investigating various hypotheses about the multifactorial pathophysiology underlying CIPN [25,28,29]. In fact, it was reported that CIPN onset involves damage to both glial and neuronal cells and related oxidative stress-mediated damage to cells [11]. In addition, cytokine activation, regulation of intracellular signaling pathways, changes of neuronal ion channel responses, impairment of axonal trafficking, and immune system activation were also investigated as putative players involved in CIPN [9] (Table 1). In any case, one of the most frequently investigated mechanisms for the pathogenesis of CIPN involves mitochondrial damage in glia and neurons [11]. Therefore, protecting mitochondrial impairment has been proposed as a promising therapeutic approach to improve clinical management of this side effect. Other studies have also shown that impairment of Ca^2+^ homeostasis—which ultimately leads to apoptosis and neuroinflammation—as possible targets for CIPN treatment [30]. Furthermore, genetic influences (including single polymorphisms) are associated with the risk of developing CIPN, although it is unclear how many actually contribute mechanistically to CIPN [8]. 

## 2. CIPN Models

Considering the high recurrence of CIPN and its limiting effect on chemotherapeutic treatment, numerous reliable animal models have been adopted to investigate it. 

Chemotherapy drugs are typically administered to mice and rats through gavage, intravenous or intraperitoneal injection. Endpoint parameters to measure CIPN are the intraepidermal unmyelinated axon density [31,32], behavioral parameters, and morphological and morphometrical analysis of neuronal soma and nerve fibers [33,34,35]. The main behavioral tests to evaluate mechanical allodynia (i.e., hypersensitivity to normally innocuous stimuli), thermal algesia and motor performance are automatic or manual von Frey, plantar, tail-flick, cold plate and rota-rod [36]. In addition to the behavioral aspect, animal models give important information at the electrophysiological level in order to characterize neurotoxicity. Therefore, nerve conduction velocity and nerve action potential are usually analyzed in the caudal and digital nerves [37]. Furthermore, the histological characterization of neuronal morphology and quantity, as well as nerve morphometry, are essential to describe axonal or myelin damage, axonal density or the distribution of axonal diameters of myelinated fibers [36]. All of these methods allow investigation of the peripheral nervous system structure changes involved in neuronopathy, axonopathy or myelinopathy [38].

Sensory neurons and their axons, but also satellite cells, are very susceptible to damage induced by antitumor treatment because of deficiency in the blood-nerve barrier, which allows easy permeation of antitumor drugs [39]. For this reason, in addition to animal models, numerous in vitro studies have been carried out, mainly on dorsal root ganglia (DRG) cultures, in order to investigate the molecular mechanisms of neurotoxicity. In fact, the DRG is the primary target of antitumor compounds causing a predominantly sensory pathology in patients treated with chemotherapy [40]. 

Different DRG-based in vitro models have been proposed in the literature: Whole DRG organotypic cultures of mice or rat embryos [41]; primary culture of (embryonal or adult) mice or rat DRG sensory neurons further treated with antineoplastic drugs [41,42]; PC12 pheochromocytoma cells [43]. In these models, the quantification of neurite outgrowth, as a functional endpoint, was used to reflect specific chemotherapy-induced neurotoxicity [44]. This parameter can be quantified with different techniques. The first method consists of a manual measure of the length of the longest neurite in each DRG by the ImageJ program [41]. A semi-automatic method, the NeuronJ plugin for ImageJ, has been developed [45]. In addition, new automatic methods have emerged based on image processing software, like ImagePro [46]. Wu and Bradshaw have used a standard phase contrast microscope to study neurotoxicity in PC12 cells [47]; Bilsland et al. have quantified, by a semi-quantitative analysis, the area occupied by neurites emanating from chick DRG explants [48]; and Popova and Jacobsson have used a fluorescent microplate assay to study βIII-tubulin immunoreactivity in P19-derived neurons [49]. Using these models, several neuroprotective substances have been tested, such as nerve growth factor (NGF) [50], as well ciliary neurotrophic factors (CNTF), brain-derived neurotrophic factors [41] or basic fibroblast growth factors [51].

In addition, ex vivo DRG cultures from adult mice treated with neurotoxic antineoplastic drugs have been used to study the mechanism underlying CIPN. This model allows preservation of a heterogeneous system, containing multiple cell types residing in a DRG, including the neurons, resident macrophages and glial cells [52]. The multiparametric morphology-centered rat DRG assay developed by Guo et al., dynamically measuring the chemotherapy-induced morphological alterations in the subcellular structures of neurons and non-neuron cells, appears to be a good model for highlighting the effect of an antineoplastic drug [53]. 

Lastly, human induced pluripotent stem cell (iPSC) derived peripheral-like neurons have often been used to predict peripheral neurotoxicants by morphological characterization and viability using high-content imaging analysis [54,55,56,57]. This approach will allow both mechanistic studies and screening of potential neuroprotective substances to prevent CIPN. The human iPSC-derived neuron represents a more favorable model than dissociated rodent neuronal culture for its high translatability to humans, due to a similar neuronal structure, which are hardly available otherwise. Therefore, in neurons derived from the iPSC model, it was demonstrated that antitumor drugs can impact neurite length independently from general cytotoxicity [54].

Moreover, some non-mammalian models have also been used to understand the mechanisms behind CIPN, such as Zebrafish or Drosophila models, but they are less usually studied [12,58,59,60]. The study of these models permits further understanding of numerous biological processes, due to similarities in many mammalian genes, rapid development with short life cycles, ready availability and easy manipulation of the genetic system. However, they cannot reproduce the complexity of the human system.

## 3. Neuronal Cytoskeleton

**Figure 1:**
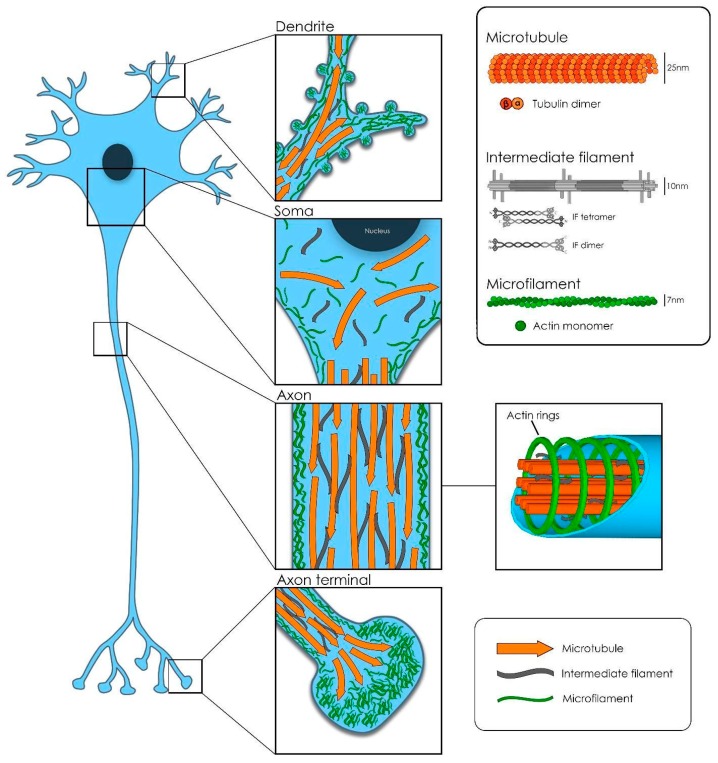
Neuronal cytoskeleton.

Successful function and development of the nervous system depend on the correct function of the cytoskeletal machinery. Cell migration, proliferation, neuron polarization and synapse network establishment are complex processes coordinated by the structural organization and dynamic remodeling of the neuronal cytoskeleton (CTK) (Figure 1). In humans, there are numerous disorders resulting from the alteration or damage of the myriad of players involved in the neuronal CTK machinery, both in the central and peripheral nervous systems. These causes vary from purely genetic to the pharmacological [61,62,63]. 

Three main polymers constitute the backbone of the elaborated and, to some extent, unknown neuronal CTK: The microfilaments, the intermediate filaments and the microtubules. However, in order to achieve proper performance, the CTK needs to associate and interact with a high number of other proteins [64].

Microtubules (MTs) are an array of α and β-tubulin heterodimers stuck together in a head-to-tail fashion, which laterally form a rather stiff, hollow tubular structure of about 25 nm diameter [65]. In neurons, bundles of MTs are kept together and stabilized by structural microtubule-associated proteins (MAP) crosslinks: Tau in axons and MAP2 in dendrites and soma. MTs are polarized structures with a plus-end and a minus-end. In axons, the plus ends are oriented towards the tip of the axon but in dendrites, MTs present a mixed polarity [66]. Another class of MAPs, called microtubule plus-end-tracking proteins (+TIPS), regulate microtubule dynamics: Polymerization, depolymerization and catastrophe phenomena. In addition, the +TIPS EB1 and EB3 recruit additional players involved in the regulation of the local axonal transport [67]. 

In fact, given that neurons are highly polarized cells, dendritic and axonal transport machinery are essential for the delivery of organelles, proteins and RNA to the distal ends (anterograde transport) in order to keep structural turnover and synaptic activity. This is also essential for the retrograde transport of aging components towards the cell body for being recycled and degraded [68,69]. In human adults for example, the length of peripheral fibers can reach 1 m, and thus the transport along axons, from the cellular body to neuromuscular junctions (or the other way around from receptors), requires a highly organized microtubule network.

The kinesin protein family is a large group of motor proteins highly expressed in neurons, where they move along microtubules in order to deliver their cargo toward the plus-end tip of the axons. In particular, motor protein kinesin-1 acts selectively, transporting cargoes in axons. This is the case even in dendrites, where it is possible to find plus-end tip microtubules distally oriented. On the other hand, cytoplasm dynein motor protein is the principal molecule responsible for retrograde transport in axons, toward the MT minus-end tip. In dendrites, instead, the unique existence of distally oriented minus-ends consents in a selective way to the minus-end-directed motor dynein to transport cargo towards dendrites tips [65,70].

One of the key mechanisms that regulate axonal transport and MT dynamics in neurons is the post-translational modification of tubulin, including as polyglutamylation, phosphorylation, polyglycylation and detyrosination. Acetylation of α-tubulin at lysine-40 by the enzyme α-tubulin acetyltransferase 1 (aTAT1) is the most studied of these modifications and is the only one that takes place in the lumen of the MT. This specific modification is present in almost all cell types and is very well conserved. Moreover, MT acetylation at lysine-40 seems to be a modification that characterizes stable long-lived MTs [71]. In fact, the absence of lysine-40 acetylation in MTs has been shown in *Caenorhabditis elegans* to lead to neuronal degeneration [72]. It has also been reported that increased acetylation could recruit and improve the docking of motor proteins to MT [73]. In addition, recent studies demonstrate that MT acetylation at lysine-40 plays an important role in mechanosensation in mammals, where sensory peripheral neurons axonal MT are highly acetylated. This modification seems to regulate the membrane stiffness of sensory neurons, tuning the ideal grade of elasticity for the best mechanical touch and pain detection [74]. 

Considering that the state of MT acetylation depends on the activity of deacetylase enzymes, some papers have investigated histone deacetylase 6 (HDAC6), an atypical cytoplasmic histone deacetylase. Unlike conventional histone deacetylase, HDAC is mostly located in the cytoplasm and has two catalytic sites. These features confer to HDAC6 the ability to interact with substrates other than histones, including α-tubulin [75,76]. The inhibition of HDAC6 has been reported to ameliorate the severity of inherited neuropathy in animal models—such as Charcot–Marie–Tooth type 2—indicating, such as mentioned above, a possible role of acetylation and HDAC6 in the onset of neuropathies [76]. Nevertheless, much still needs to be elucidated about the role of post-transcriptional modifications on MT function and dynamics.

Two other essential bricks constitute the complex CTK structure: The microfilaments of actin and the intermediate filaments or neurofilaments. All of them combined, form a stable and functional network of polymers that are tightly associated and work in synergy. 

Actin monomers polymerize forming a double helical structure, thinner (about 7nm) and more flexible than the MTs. In the periphery of the neuronal body, attached to the inner plasma membrane through anchoring proteins (ERM proteins), actin filaments form a meshwork together with actin-binding proteins and myosin motors. This so-called actomyosin cortex has an important role in protecting the cell shape against mechanical stress and in cell shape control [64]. In axons, however, actin ring-like structures bind to the inner membrane of the axon and are periodically arranged (~180 to 190 nanometers) along their shaft. In this way, MTs and actin jointly create a strong structure, able to resist the mechanical deformation forces exerted, in particular, on those axons that need to cover long distances (from hundreds of micrometer up to 1 m, depending on the cell type and on the species). Moreover, periodic actin ring organization has a very important role in stabilizing MT remodeling in growing and mature axons. In dendrites, instead, long actin filaments are positioned along the shaft [77,78]. The actin cytoskeleton is also responsible for the maintenance of dendritic spines shape, as well as for their dynamic morphological changes. The crosstalk of actin with dendritic MTs, together with the presence of some associated proteins (drebrin, end-binding EB3 or cortactin-binding protein 2), regulates the shape dynamics in spines during spinogenesis and throughout learning and memory processes [64,79]. During development, at the distal end of the incipient processes, complex networks of actin and a high number of actin-associated proteins work together with MTs to construct the polarized mature neuron. This complex machinery helps the exploratory filopodia and lamellipodia to detect the surrounding attractive or repellent cues present in the environment that will determine, in the end, the direction and the speed of the growing neurite until the final formation of a new synapse [80,81]. Therefore, MTs and actin must interact and be physically and functionally tightly coupled for the correct steering of axon and dendrite growth, spine plasticity and synapse formation [82]. 

An additional vital component of the cellular cytoskeleton is the intermediate filament. There are six major classes expressed in different cell types, all with a similar basic structure [83]. In neurons, neurofilaments (NF) constitute the class IV of intermediate filaments and are extremely long-lived proteins. NFs are 10 nm diameter twisted protein strands, whose composition (the relative subunit expression levels) varies at each neuron type at a specific stage of development. In fact, NFs are a combination of four different subunits: Light (NF-L), medium (NF-M) and heavy (NF-H) polypeptides, and the proteins α-internexin (present in the central nervous system) or peripherin (present only in the peripheral nervous system, or PNS). All three-polypeptide subunits differ highly on their aminoacidic composition at their head domain (amino-terminal end) and in particular, at the tail domain (carboxy-terminal end), which is also very variable in length. In addition, the presence of more or less post-translational modifications (mainly phosphorylation and glycosylation) contribute to the heterogeneity of these subunits [84,85]. NF structure is formed of the parallel side by side association of polypeptide dimers that in turn, arrange into staggered antiparallel tetramers. This particular organization of the NF tetramers produces a construct void of polarity, in contrast with the polar structures of actin filaments and MTs. The lateral association of eight tetramers creates cylindrical structures called unit length filaments, which join end-to-end to generate the final NF structure [83,86].

Their flexible fibrous structure allows the neuron to maintain its markedly asymmetric shape [87]. Neurofilaments are the most abundant CTK component in large myelinated caliber axons (with respect to perikarya and dendrites), where they have a role in developing and maintaining the three-dimensional array of the axoplasm. Moreover, neurofilaments are required for axon radial growth [88,89]. The carboxyl terminal of the filaments is subject to different post-translational modifications and protrude radially from the main filamentous structure. In particular, the side arms of the NF-M and NF-H subunits are responsible for keeping distant the neighboring filaments, in a degree that determines the diameter of the axon and, consequently, its final conduction velocity properties [83,90,91].

As already mentioned, the interaction of the three components of the cytoskeleton is fundamental for neuronal structural organization. Consequently, the proteins that allow the crosstalk among them are crucial, and present a possible direct or indirect target for neurotoxic drugs or neurological disease.

Spectraplakins are a family of large intracellular proteins with the peculiarity of being able to associate to all three filamentous components of the CTK [92,93]. In particular, dystonin (also known as bullous pemphigoid antigen 1, or BPAG1), is a very large spectraplakin protein present in different tissues such as the nervous system, skin and muscle. In the PNS, it is expressed primarily in sensory neurons, such as those of the DRG. Dystonin is essential for maintaining cytoskeletal organization and stability, organelle integrity, and intracellular transport. This protein is associated with the cytoskeleton, forming a bridge between F-actin and the intermediate filaments [94]. Loss of dystonin function causes dystonia musculorum (dt) in mice, a progressive loss of limb coordination that is the consequence of severe CTK disorganization in sensory neurons of the DRG [95]. Specifically, in DRG, there are several known subunits: Dystonin a1, a2 and a3. They present different N-terminal domains and different cellular distributions. For example, Dyst-a1 (also known as BPAG1n4) is present in axons, where it seems to be involved in retrograde transport mediated by dynactin [96,97]. Dyst-a2 possesses a transmembrane domain and has been found anchored to the external nuclear membrane [98], where it plays a role in perikaryon CTK organization, endoplasmic reticulum (ER) structure organization and endoplasmic reticulum-Golgi apparatus (ER-Golgi) vesicular transport [99]. Dyst-a3, however, localizes close to the plasma membrane [100].

## 4. Microtubules and CIPN

Considering the high compartmentalization of neurons and the central role of microtubules in cargo trafficking, most studies about neurotoxicity have focused on this class of cytoskeleton polymer [101,102,103,104,105,106]. Moreover, several neurodegenerative diseases of the peripheral nervous system present MT injury [107,108]. Microtubule function and/or structural perturbation also includes those affecting MAPs or other proteins able to interact with tubulin monomer or polymer.

### 4.1. Axonal Transport 

As mentioned above, axonal transport allows the maintenance and restock of nerve endings with proteins, lipids and organelles (especially mitochondria). It also removes misfolded or damaged proteins that should be recycled [109,110]. 

Among all hypothesis proposed in the literature, impairment of axonal transport is one of the most plausible mechanisms that could lead to MTA-induced CIPN. Even if it is still unclear how these drugs alter axonal transport, it is known that they do not interfere by steric obstruction. For example, the binding site of taxanes and epothilones is localized on the inner surface of the MT, while motor proteins dock and move along the outer surface [111]. It is likely that the binding of both drugs induces a conformational change in the MT that alters the affinity between tubulin and motor proteins or other non-motor MAPs [112,113]. 

La Pointe et al. have evaluated the different effect of four main different classes of MTA (vincristine, eribulin, paclitaxel and ixabepilone) on axonal transport using vesicle the transport/squid axoplasm assay and the MT gliding assay [17]. They have hypothesized that the severity of neuropathy induced by these drugs is directly correlated with their ability to impair axonal transport. They have demonstrated that vincristine and ixabepilone reduce both anterograde and retrograde fast axonal transport, affecting also kinesin-1 velocity, while paclitaxel and eribulin impair only anterograde transport in a less significant manner and without affecting kinesin-1 movement. 

The differences in neurotoxicity observed between paclitaxel and ixabepilone could be explained by their binding affinity. They are both MT-stabilizing agents, but ixabepilone binds to tubulin stronger than paclitaxel [114,115]. Moreover, their binding site to tubulin is not identical and for this reason, they could induce a different alteration in conformation and tubulin post-translational modification, especially in the c-terminal, a site that is important for motor proteins and MAP interaction and regulation [111,116,117]. 

Vincristine and eribulin are both MT destabilizing agents, but they impair axonal transport with different intensities. This difference could be also in this case explained by their different binding site: Eribulin binds only the plus-end of MT and exerts its destabilizing effect only here [118]. Conversely, vincristine binds along the entire MT and its effect destabilizes all the structure, altering the conformation of the MT and the interaction with motor proteins and MAPs [14,113].

### 4.2. Microtubule Polarity

As mentioned above, MT orientation is mixed in the cellular body and in dendrites, while it is highly polarized in axons. Here, MTs have the minus-end facing the cellular body and the plus-end facing the synapses. Alteration in this organization could lead to impaired axonal transport and consequently, axon degeneration and a decrease in conduction velocity [102,119,120]. In fact, Shemesh et al. have demonstrated that paclitaxel induces the reconfiguration of the MT from a highly polar oriented MT to a chaotic distribution [121]. This could be one of the possible causes of fast axonal transport impairment. How paclitaxel induces this alteration is still unclear, but two main hypotheses have been formulated. (1) Paclitaxel induces conformational changes that curve the MT in a loop. Until now, however, this alteration has been only observed in tubulin polymerization assays, but not in cell cultures or in vivo animal models [121,122,123]; (2) paclitaxel causes the formation of new nucleation sites that sequester tubulin dimers, from which newly assembled MTs extend in various directions [124]. Several research groups have also demonstrated the importance of MT conformation. In particular, a GTP/GDP (guanosine triphosphate/guanosine diphosphate) ratio alteration could induce conformational changes in the curvature of the MTs. In fact, the binding affinity of several MAPs and motor proteins is different on the base of MT curvature. For example, Nakata et al. have demonstrated that KIF5 (a kinesin-1) preferentially binds to GTP-rich regions of MT [125].

### 4.3. Microtubule-Associated Proteins 

Microtubule-associated proteins (MAPs) are a class of proteins that interact with tubulin and allow MT to carry out all their functions. Paclitaxel impairment of axonal transport could be due to alteration of MAPs, or regulatory protein (for example kinases) expression or post-translational modifications [126,127,128]. Kinesin and dynein post-translational modifications have been reported in several inherited neurodegenerative diseases and this could be a valid mechanism in MTA-induced CIPN. One of the most studied modifications of kinesin is phosphorylation by JNK (c-Jun N-terminal kinase). This phosphorylation reduces the activity of kinesin. Moreover, the activation of JNK has been reported in non-cancer cells and in cortical neuron cultures after paclitaxel treatment [126,129]. This could explain the reduced axonal transport observed in paclitaxel-induced neurotoxicity. 

Several other protein kinases regulate axonal transport through direct phosphorylation of motor proteins, adapter and cargos. Moreover, the dysregulation of axonal transport by kinases has been implicated in the pathogenesis of several neuropathies [130,131,132]. Both kinesin and dynein are regulated by protein kinases such as GSK3β, ERK1/2, JNK and Akt [127]. GSK3β activation induces the phosphorylation of kinesin and dynein motor proteins, reducing their movement along the MT. Interestingly, Gao et al. have demonstrated that paclitaxel increased activation of GSK3β in the spinal dorsal horn of rats and that paclitaxel-induced neuropathy is prevented when animals receive lithium treatment (a GSK3β inhibitor) [133]. 

As mentioned above, Tau is localized—particularly in the axons of mature neurons—and has been widely investigated due to its key role in neuronal development and in many neurodegenerative diseases [134,135,136]. Tau has two binding sites on the MT and one of these sites partially overlaps with the binding site of paclitaxel. Tau and Paclitaxel both suppress dynamic instability and increase stabilization of the MT, but Choi et al. have also demonstrated that they modulate the diameter of the MT and change the number of protofilaments by different amounts [137]. It is possible that, due to the overlapping binding site, paclitaxel competes with Tau for docking to the MT and induces a different conformational change in MT curvature and diameter. Samsonov et al. have shown that treatment with Paclitaxel reduced the ratio of Tau bound to the MT, confirming the competition to the same binding site [138]. Moreover, Park et al. have reported that polymorphisms in Tau-associated genes (i.e., GSK3β) may contribute to the development of paclitaxel-induced neuropathy [139]. They found a GSK3β polymorphism that increases Tau phosphorylation, reducing the rate of Tau association with the MT. Consequently, the MTs are less stable and more sensitive to paclitaxel. This could explain the role of tau phosphorylation in axonal damage and neuropathy induced by paclitaxel. 

MAP1B is an MT-associated protein with important roles in development (axonal guidance and elongation) and function of the nervous system. In mature neurons, it participates in the regulation of the structure and physiology of dendritic spines. It binds to two subunits of tubulin and is involved in the nucleation and stabilization of the MT. It is reported that mutations or a knockout of the MAP1B gene change EB1 and EB3 interaction with the MT, leading to altered dynamics, directionality and curvature of the MT [140]. Brazil et al. have optimized a model of paclitaxel-induced peripheral neuropathy using *Drosophila* larvae and demonstrated that nociceptive sensitivity was associated with the disrupted organization of MAP1B/Futsch and an aberrant stabilization of peripheral sensory dendrites of class IV dendritic arborization nociceptors [141]. 

### 4.4. End-Tracking Proteins

Plus-end tracking proteins (+TIPs) are fundamental for the promotion and the stabilization of the growing MT [142]. +TIPs also have a central role in the interactions that occur between microtubules and actin [143,144,145]. 

Eribulin binds directly to the plus-end of the MT, its binding site is probably partially overlapping with some +TIP sites, and this could interfere with MT stability. O’Rurke et al. have demonstrated that the treatment with eribulin induces a dose-dependent reduction in the binding of the +TIP EB1 to the MT. The consequence was a reduction of MT stability and increased depolymerization [118]. 

Taxanes and vinca alkaloids also modulate the dynamics of MT plus-ends and disrupt EB protein localization. However, very little is known about the MTA action on +TIPs. Tubulin and microtubule conformational changes induced by taxanes and vinca alkaloids may alter +TIP interactions with tubulin or decrease the available EB binding sites at the microtubule plus-ends. Alternatively, MTAs could regulate EB-microtubule interaction indirectly via specific changes on EB protein phosphorylation [146].

Recently, Rovini et al. have demonstrated that paclitaxel treatment induces the loss of EB3-comet tails in cultured *Aplysia* neurons, with a subsequent reduction in MT dynamics and impaired axonal transport [146]. They have also reported that olesoxime partially reverts this alteration, preventing the delocalization of EB1 and EB3 proteins and preserving the MT dynamics. 

### 4.5. Post-Translational Modification

In recent work, Van Helleputte et al. have demonstrated that the inhibition of histone deacetylase HDAC6 increases the acetylation of tubulin in several tissues of rodents and reduces the severity of the neuropathy induced by vincristine [147]. Increased tubulin acetylation induced by HDAC6 inhibitors partially restores MT stability and axonal transport. Moreover, tubulin acetylation increased the interaction and docking of motor proteins [76,147].

Similarly, Krukowski et al. demonstrated the same neuroprotective effect in an animal model treated with cisplatin [148]. The inhibition of HDAC6 increased α-tubulin acetylation in peripheral nerves, restoring axonal transport and mitochondrial function, and reverting mechanical allodynia induced by cisplatin. 

### 4.6. Other Possible Microtubule-Correlated Pathways 

Heat shock protein 27 (Hsp27) is involved in the reduction of reactive oxygen species during oxidative stress, anti-apoptotic activity under condition of chemical stress, and protein folding, but also interacts with actin and the MT [119]. The interaction with the MT is still poorly understood, but Hsp27 participates in the stabilization of the MT [149,150]. The role of Hsp27 in neurodegeneration has been reported in some inherited diseases. For example, mutations of HSP27 genes, inducing its higher affinity binding to the MT and consequently MT stabilization, are reported to cause Charcot–Marie–Tooth neuropathy [151]. A research group demonstrated that the overexpression of Hsp27 protects from paclitaxel-induced neuropathy. They proposed a different hypothesis, where a possible Hsp27 direct effect on the MT exists [119].

Mitofusins (Mfn1 and Mfn2) are proteins localized in the outer mitochondrial membrane and are involved in the fusion process of mitochondria. They are also involved in mitochondria transport along axons. Indeed, Mfn1 and Mfn2 interact with Miro1/Miro2 and the Milton proteins to form a complex that link mitochondria to kinesin motor proteins [152,153,154]. Mutations in *Mfn2* cause Charcot–Marie–Tooth (CMT) type 2A, a disease characterized by degeneration of peripheral axons [155,156]. Bobylev et al. have demonstrated that cisplatin induces a reduction in the expression levels of Mfn2 [157]. Since cisplatin-induced peripheral neuropathy is linked to mitochondrial damage and alterations in their transport, it is possible that a reduction in Mfn2 expression could lead to impairment of mitochondria axonal transport. Yamashita et al. have also hypothesized that paclitaxel may induce peripheral neuropathy, due to changes in Mfn2 expression [158]. They have demonstrated that rats treated with paclitaxel show a reduction in Mfn2 expression levels in the spinal cord. This reduction is observed before the appearance of mechanical allodynia, suggesting that a reduction in Mfn2 expression contributes to paclitaxel-induced mechanical allodynia. 

NF-κB is a transcriptional regulator that is physiologically inhibited by inhibitor kB alpha (IkBα). The activation of NF-κB occurs when the proteasome degrades IkBα and NF-κB translocates into the nucleus. In cancer cells, Bortezomib (BTZ) inhibits proteasome degradation of IkBα and, consequently NF-κB activation [159]. However, in neurons the situation is different. Alé et al. demonstrated that BTZ treatment induces the activation and nuclear translocation of NF-κB and consequently the increase of pro-inflammatory cytokines synthesis and cellular activation [22,160,161]. Pharmacological inhibition of NF-κB in neuron cultures reduces the degeneration of axons induced by BTZ and transgenic mice overexpressing IkBa suffer a less severe BTZ-induced neuropathy (BIPN). These data demonstrate a key role of NF-κB in BIPN, but the mechanism of NF-κB activation in neurons is still unclear. Several research groups have reported a correlation between the alteration in MT polymerization or axon damage and NF-κB activation, with a consequent increase in pro-inflammatory cytokines and inflammation [162,163,164]. 

## 5. Intermediate Filaments and CIPN

Because of the central role of intermediate filaments in maintaining cell integrity, axonal growth and caliber, and plasticity of the cytoskeleton, and considering their involvement in neurodegenerative disorders [165], these components of the cytoskeleton have been investigated in several papers regarding neurotoxicity. In the literature, there are data regarding expression, post-translational modifications and altered distributions of intermediate filaments. Meyer et al. have investigated, in three different papers, the effect of vincristine [166], oxaliplatin [167] and paclitaxel [168] on NF-H expression, studying also the protective effect of neurosteroids. Vincristine induced disorganization and a 44% decrease of NF-H immunostaining in rat sciatic nerves [166]. Interestingly, neurosteroids are able to reverse (to normal values) the vincristine-induced, decreased levels of NF-H, together with nerve conduction velocity, pain transmission abnormalities and intraepidermal nerve fiber density. It is important to underline that treatment of naïve rats with neurosteroids does not increase NF-H levels, suggesting that the effect of neurosteroids on NF-H may be indirect and that neurosteroids could affect the expression or activation levels of intracellular factors altered by vincristine. 

On the other hand, Meyer et al. have highlighted that oxaliplatin induced a decrease of NF-H immunoreactivity in a rat sciatic nerve (59%) or the lumbar DRG (48%), together with sciatic nerve conduction velocity and peak amplitude reduction [167]. All these effects were reversed by allopregnanolone. This strict correlation between NF-H expression and electrophysiological parameters is in agreement with data obtained in each NF knock-out mouse [169]. In particular, Kriz et al., have demonstrated that low expression of NF-H induces axonal diameter reduction and consequently nerve conduction velocity slows down. In addition, Jamieson et al. have investigated the effect of different antineoplastic drugs on phosphorylated neurofilaments heavy chain (pNF-H) in the L5 dorsal root ganglia of adult female Wistar rats [170]. Oxaliplatin treatment induces a reduction of pNF-H immunoreactivity in neuronal cell bodies but it does not affect NF-H phosphorylation or expression in nerve fibers. Moreover, it is noteworthy that this endpoint is tightly correlated with the neurotoxicity of oxaliplatin, cisplatin and carboplatin. Conversely, it is not related to paclitaxel-induced neurotoxicity, suggesting that the reduction of pNF-H could be caused by an inhibition of NF-H kinase gene transcription (or induction of NF-H phosphatase), which is determined in a specific way by platinum-based drugs.

Paclitaxel (1 mg/kg for 7 days treatment), like vincristine and oxaliplatin, reduces (33%) NF-H immunostaining in treated rat sciatic nerve axons [168]. Paclitaxel-induced neuropathy and NF-H expression reduction is prevented by the natural neurosteroid 3α-androstanediol. Similarly to the effect of neurosteroids on vincristine-induced NF-H expression modulation, 3α-androstanediol also seems to modify the activation of transcription factors evoked by paclitaxel treatment. No paclitaxel-induced alterations of pNF-H expression in DRG neurons have been shown by Jamieson et al. [170].

Moreover, Alè et al. have demonstrated an alteration of neurofilament organization induced by bortezomib, both in in vitro and in vivo models [171]. In particular, in DRG neurons treated with 4 nM bortezomib, sensory neurons showed disruption of NF-H staining along neurites, whereas soma are characterized by their accumulation. DRG ganglia explanted from bortezomib-treated (2 mg/kg) mice show no alteration of NF-H mRNA expression, but an increase of the non-phosphorylated form of NF-H.

It is noteworthy that phosphorylation state of NFs is fundamental because this post-translational modification can perturb their own axonal transport, and consequently their distribution in neurons. In fact, many studies demonstrated that the rate of NF axonal transport is inversely correlated to their phosphorylation state [172]. In particular, C-terminal NF phosphorylation decreases the affinity for kinesin (anterograde transport) and increases their affinity for dynein (retrograde transport), whereas NF hypophosphorylation improves their anterograde dynein-mediated transport, as cargo of those microtubules are being translocated along axons [173]. At the same time though, NF phosphorylation could improve the NF-NF interaction, possibly making an NF macrostructure too large to be transported in retrograde. Moreover, the proper aggregation and distribution of neurofilaments can be modified by alteration of molecular motors or malfunction of microtubule dynamic structure, as already mentioned.

## 6. Microfilaments and CIPN

Surprisingly, a very small number of studies dealing with CIPN consider the effect of chemotherapeutics on microfilaments, despite their central role in maintaining the morphology of neurons and the numerous proteins with which they interact both in actin rings, actin waves, actin trails and hotspots [174].

Considering the little data present in the literature, the effect of antineoplastic drugs on microfilaments, and in particular, on actin rings and actin waves, should be deeply studied, considering the wide actin-microtubule crosstalk [64]. In fact, microfilaments or actin-binding proteins may be a target of antineoplastic drugs, which ultimately affect the rate of axonal microtubule bundling. Burnette et al. have indeed demonstrated that the actin ring in the initial axon segment, interacting with myosin, can align and promote the formation of the microtubule bundle [175]. Moreover, crosstalk between microfilaments and microtubules appears increasingly important in determining axon specification and outgrowth and in maintaining axonal mechanical protection.

James et al. have demonstrated in vitro, that paclitaxel and Cisplatin (CDDP) induce neurite degeneration, which affects the arrangement of the actin cytoskeleton in several neurons models, including DRG sensory neurons [176]. All the antineoplastic drugs tested reduced neurite outgrowth and branch number but, in particular, cisplatin neurotoxicity is mitigated by the reduction of RhoA activity. RhoA belongs to the Rho family small GTPases, which are regulatory molecules that link surface receptors to actin, as well as to microtubule cytoskeleton, and thus play a fundamental role in regulating neuronal morphology, dendritic arborization, spine morphogenesis and growth cone development [177]. It was demonstrated that: Rho inhibition improves axonal outgrowth and regeneration after injury [178] and; the Rho GTPases are involved in some neurodegenerative diseases [179]. 

Karademir et al., investigating the possible causes of different CIPN—induced by the two proteasome inhibitors BTZ and Carfilzomib (CFZ)—using a proteomic approach in a neural stem cell model, have demonstrated that among the most affected proteins (including microtubule) are actin and the actin-binding proteins [180]. In particular, BTZ and CFZ induce an actin density decrease, directly dependent on their neurotoxicity. Moreover, BTZ and CFZ are able to induce a transitory 2-fold increase of actin-related protein 2 (Arp2) expression, a protein that controls actin patch precursors in the PNS [181]. The increase of Arp2 expression after proteasome inhibitor treatment could be interpreted as a cellular response vs the destabilization of actin filaments. Moreover, interestingly BTZ but not CFZ increases some heat shock protein levels, including Hsp70, which interacts with actin and is involved in its transports to the proteasome [182].

In addition, in primary sensory neurons purified from Hsp27 transgenic mice, the overexpression of Hsp27 was proven to be effective in protecting from paclitaxel-induced neurite outgrowth inhibition [119]. Hsp27 is known to be an actin capping protein, able to bind to the terminal part of actin filaments, regulating their polymerization in several cell types. The same neuroprotection has been demonstrated in an in vivo experiment. Data suggest that the interaction between Hsp27 and the F-actin tips in the DRG could accelerate axonal growth and promote recovery from peripheral nerve damage. In this model, the Hsp27-induced axonal promoting effect could be correlated to inhibition of RhoA activity, as already demonstrated in rat cortical neurons [182].

## 7. Conclusions

Despite the development of new and more effective chemotherapies, most of the antineoplastic drugs used show a neurotoxic effect, that is dose-limiting and often determines discontinuation of treatment [1]. Furthermore, it is clear that the different classes of drugs, despite having a different antineoplastic effect, often induce CIPN characterized by similar and principally sensorial symptoms. At present, none of the strategies proposed, including complementary therapies with medicinal plants recently suggested [183], have proven effective in preventing or reducing nerve damage induced by antineoplastic drugs [1,27]. Starting from these concepts, this review has been focused on the changes induced by different chemotherapies on the neuronal cytoskeleton, a dynamic and diversified backbone in each neuron district, which is fundamental for neuronal structure and influences (with all three its components) all neurons functions. It is relevant to underline, that most of the papers regarding CIPN pathways are focused, independently of the chemotherapy drug under study, on the effect of antineoplastic agents on axon transport mediated by microtubules (which can perturb the organization of the other components of the cytoskeleton). From the data collected in this review, it is evident that there are some antineoplastic drug-induced effects that result independent of this mechanism. Tacchetti et al. studied the gene networks and functions of differentially expressed genes in the plasma cells of patients who developed bortezomib-thalidomide-dexamethasone-induced peripheral neuropathy, and demonstrated significant deregulation of processes involved in cytoskeleton rearrangement [184]. 

In order to study CIPN, cytoskeleton-related pathways that should be investigated are countless, considering the very high number of cytoskeleton binding proteins and of post-translational modifications of the MT, NF and microfilaments. In this context, taking into account the importance of crosstalk among the three components of the cytoskeleton, studies should focus on this aspect.

As mentioned in this review, several papers reported chemotherapy-induced modifications of the distribution of neurofilaments and of phosphorylation, in particular, of NF-H. However, in the literature, there is a lack of data regarding the activity of related kinases and phosphatases in CIPN models. The relevance of the C-terminal region of NF-H in mediating the interactions of NFs and other cytoskeleton components is well demonstrated [185,186,187]. Considering the fundamental role of neurofilaments in maintaining the stability of the axon (in particular, in determining the axon caliber in large fibers), in the future, it would be desirable to understand the effect of different classes of antineoplastic drugs on the mechanisms through which NF-H phosphorylation occurs, and the resulting effects on the other cytoskeleton components.

Based on the data in the literature regarding MT post-transcriptional modification, we think that alteration of MT lysine-40 acetylation is worthy of further study. In fact, it is a highly conserved modification, particularly present in the sensory neurons in mice, where it seems to modulate mechanosensation, among other functions. It is noteworthy to remark that MT acetylation has been reported relevant in a series of neuronal diseases, such as Alzheimer’s disease (AD), Parkinson’s disease (PD), Rett syndrome and Charcot–Marie–Tooth (CMT) disease [188], while only some papers report a link between MT acetylation and CIPN. Moreover, in this context, only an indirect correlation of HDAC6 activity and CIPN has been studied. However, there are very few in vivo reports investigating the possible interaction between chemotherapy drugs and TAT1 activity. This interaction has been investigated only in vitro by Kalebic et al. [189].

The effect of antineoplastic drugs on microfilaments, still scarcely investigated, could be a very interesting goal. Despite the report that the actin ring, along the axon, stabilizes microtubule bundles, the pathway by which this phenomenon occurred is still not elucidated. Future CIPN studies should be focused on this aspect. In particular, spectraplakins could be an interesting target for neuroprotective studies, considering their ability to bind to all three components of the cytoskeleton. The importance of the study of the effect of chemotherapeutic agents on actin is even more evident, considering that in vitro, different classes of antineoplastic drug induce not only the reduction of neurite outgrowth but also the increase of neurite arborization (author’s personal observation), an event strictly modulated by actin filaments in growing neurites [190].

Finally, in the future, it would be desirable to develop pharmacological strategies based on a more in-depth knowledge of the effects that the different chemotherapeutic agents have on the ability of the three components of the cytoskeleton to crosstalk among themselves.

## Figures and Tables

**Table 1:** Summary of antineoplastic and neurotoxic mechanisms in chemotherapy-induced peripheral neurotoxicity. DRG: Dorsal root ganglia; NGF: Nerve growth factor; TRP: Transient receptor potential channels.

Chemotherapy Agent	Antineoplastic Mechanisms	CIPN Pathophysiology
**Taxanes**	Microtubule damage that impairs mitotic spindle formation in cancer cells [4,5,6].	Activation of caspases, oxidative stress on peripheral neuronal and non-neuronal cells, mitotoxicity, inhibition of anterograde fast axonal transport, prevention of microtubule disassembly, alteration of both activity and expression of voltage-gated ion channels in the DRG; immune activation in the DRG and peripheral nerves, and microglial activation in the spinal cord; TRP upregulation in DRG [7,8,9,10,11,12].
**Vinca alkaloids**	Binding to free tubulin dimers close to the GTP-binding sites and induction of cell death by inhibition of microtubule assembly [13,14].	Increase of microtubule depolymerization and inhibition of the hydrolysis of GTP; membrane excitability, inflammation, axonal transport impairment; mitochondria and glial function alterations; differential expression of voltage-gated ion channels, alteration of neurotransmission, impairment of axonal transport, increased production and release of proinflammatory cytokine and chemokines [8,9,11,12,15].
**Eribulin**	Suppression of microtubule dynamic instability at low concentration and promotion of microtubule disassembly at high concentration [16].	Reduction of anterograde fast axonal transport; reduction of kinesin-dependent transport in axon [17].
**Epothilones**	Stabilization of microtubules, leading to apoptosis in cancer cells [18].	Microtubule stabilization; reduction of kinesin-dependent transport [17].
**Platinum compounds**	Platinum-DNA adduct formation, alterations in transmembrane receptors and channels that lead to cell cycle arrest and apoptosis [19,20].	Accumulation of platinum atom in the DRG sensory neurons, nuclear and mitochondrial DNA damage, oxidative stress and channellopathy; TRP channels affected; axonal transport impairment; activation of caspases and protein kinase, glial cell activation, increased production and release of proinflammatory cytokine and chemokines [7,8,9,11,12,21].
**Proteasome inhibitors**	Inhibition of proteasome activity, which results in protein aggregate accumulation in tumor cells, cell cycle arrest and apoptosis [22,23].	Protein aggregate accumulation in soma neurons, alteration of physiological turnover of axonal proteins, axonal transport impairment; damage to neuronal mitochondria and organelles and Schwann cell microtubule stabilization, oxidative stress; activation of TRP channels; inflammation and glial cell activation; alterations of neurotransmission [9,10,11,12,15].
**Thalidomide**	Immunomodulation and antiangiogenic effects, down-regulation of tumor necrosis factor alpha (TNFα) [24].	Capillary damage due to its antiangiogenic activity in nerve fibers; downregulation of TNFα and inhibition of NF-κB that interferes with NGF activity [10,21].
